# The Campo de Dalias GNSS Network Unveils the Interaction between Roll-Back and Indentation Tectonics in the Gibraltar Arc

**DOI:** 10.3390/s22062128

**Published:** 2022-03-09

**Authors:** Jesús Galindo-Zaldivar, Antonio J. Gil, Víctor Tendero-Salmerón, María J. Borque, Gemma Ercilla, Lourdes González-Castillo, Alberto Sánchez-Alzola, María C. Lacy, Ferran Estrada, Manuel Avilés, Pedro Alfaro, Asier Madarieta-Txurruka, Fernando Chacón

**Affiliations:** 1 Departamento de Geodinámica, Universidad de Granada, 18071 Granada, Spain; lgcastillo@ugr.es (L.G.-C.); amadatxu@ugr.es (A.M.-T.); 2 Instituto Andaluz de Ciencias de la Tierra (CSIC-UGR), 18071 Granada, Spain; vtendero@ugr.es; 3 Departamento Ing. Cartográfica, Geodésica y Fotogrametría, Universidad de Jaén, Campus de Las Lagunillas, 23071 Jaén, Spain; ajgil@ujaen.es (A.J.G.); mjborque@ujaen.es (M.J.B.); mclacy@ujaen.es (M.C.L.); maviles@ujaen.es (M.A.); fchacon@ujaen.es (F.C.); 4 Centro de Estudios Avanzados en Ciencias de la Tierra, Energía y Medio Ambiente (CEACTEMA), Universidad de Jaén, Campus de Las Lagunillas, 23071 Jaén, Spain; 5 Instituto de Ciencias del Mar, CSIC, Continental Margins Group, 08003 Barcelona, Spain; gemma@icm.csic.es (G.E.); festrada@icm.csic.es (F.E.); 6 Departamento de Estadística e Investigación Operativa, Universidad de Cádiz, 11510 Puerto Real, Spain; alberto.sanchez@gm.uca.es; 7 Departamento de Ciencias de la Tierra y del Medio Ambiente, Facultad de Ciencias, Universidad de Alicante, 03080 Alicante, Spain; pedro.alfaro@ua.es

**Keywords:** GNSS network, active fold and fault interaction, roll-back, indentation tectonics, Gibraltar Arc

## Abstract

The Gibraltar Arc includes the Betic and Rif Cordilleras surrounding the Alboran Sea; it is formed at the northwest–southeast Eurasia–Nubia convergent plate boundary in the westernmost Mediterranean. Since 2006, the Campo de Dalias GNSS network has monitored active tectonic deformation of the most seismically active area on the north coast of the Alboran Sea. Our results show that the residual deformation rates with respect to Eurasia range from 1.7 to 3.0 mm/year; roughly homogenous west-southwestward displacements of the northern sites occur, while the southern sites evidence irregular displacements towards the west and northwest. This deformation pattern supports simultaneous east-northeast–west-southwest extension, accommodated by normal and oblique faults, and north-northwest–south-southeast shortening that develops east-northeast–west-southwest folds. Moreover, the GNSS results point to dextral creep of the main northwest–southeast Balanegra Fault. These GNNS results thus reveal, for the first time, present-day interaction of the roll-back tectonics of the Rif–Gibraltar–Betic slab in the western part of the Gibraltar Arc with the indentation tectonics affecting the eastern and southern areas, providing new insights for improving tectonic models of arcuate orogens.

## 1. Introduction

GNSS networks make it possible to determine the present-day strain field, thereby contributing to our overall understanding of active tectonic processes [1]. Regional networks provide valuable new data on plate motion [2], whereas local networks focus on the most active tectonic structures, mainly formed at plate boundaries. The networks may characterize the fold [3] or fault activity as having creep or seismic behavior [4]. Recent geological evolution derived from field geological and geophysical studies is completed through analysis of the active tectonic deformation provided by geodetic data [5]. Moreover, in coastal areas, the seaward continuity of tectonic structures offers a stage to integrate complementary—offshore and onshore—geological observations [6].

**Figure 1:**
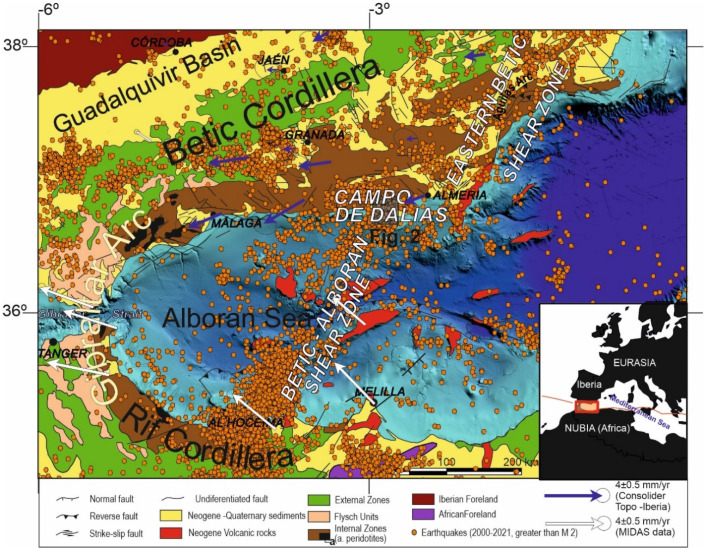
Regional geological setting and seismicity of the Gibraltar Arc: Major geological units are included onshore. Offshore, the main tectonic structures are indicated with detailed bathymetry. Earthquake data from IGN (www.ign.es, accessed on 1 February 2022); GNSS displacement in the central and eastern Betic Cordillera from the Consolider Topo-Iberia network [13,14,15], and in the Rif Cordillera, Gibraltar Strait, and central Alboran Sea from MIDAS velocity fields, Nevada Geodetic Laboratory (http://geodesy.unr.edu/velocities/midas.IGS14.txt, accessed on 20 February 2022). The location of the Campo de Dalias study area (Figure 2) is indicated.

The Gibraltar Arc (Figure 1) is an Alpine arched tectonic belt developed since the Cenozoic at the Eurasia–Nubia (Africa) plate boundary [7], currently undergoing a regional northwest–southeast convergence rate of 4.5 to 5 mm/year [8,9,10,11,12]; it formed as a result of the Betic Cordillera’s connection through the Strait of Gibraltar to the Rif Cordillera, surrounding the Alboran Sea, in the westernmost Mediterranean Sea.

In 2006, we installed a non-permanent GNSS network in the western Campo de Dalias [16]. The network was initially intended to quantify the activity of the northwest–southeast normal Balanegra Fault—one of the most active faults in the area—related to the 1993–94 seismic series. The throw of the Balanegra Fault determined the straight orientation of the coastline, separating the northeast uplifted Campo de Dalias block from the southwest downthrown block, located below sea level. Vertical deformation rates obtained from high-precision leveling lines were analyzed by [17].

**Figure 2:**
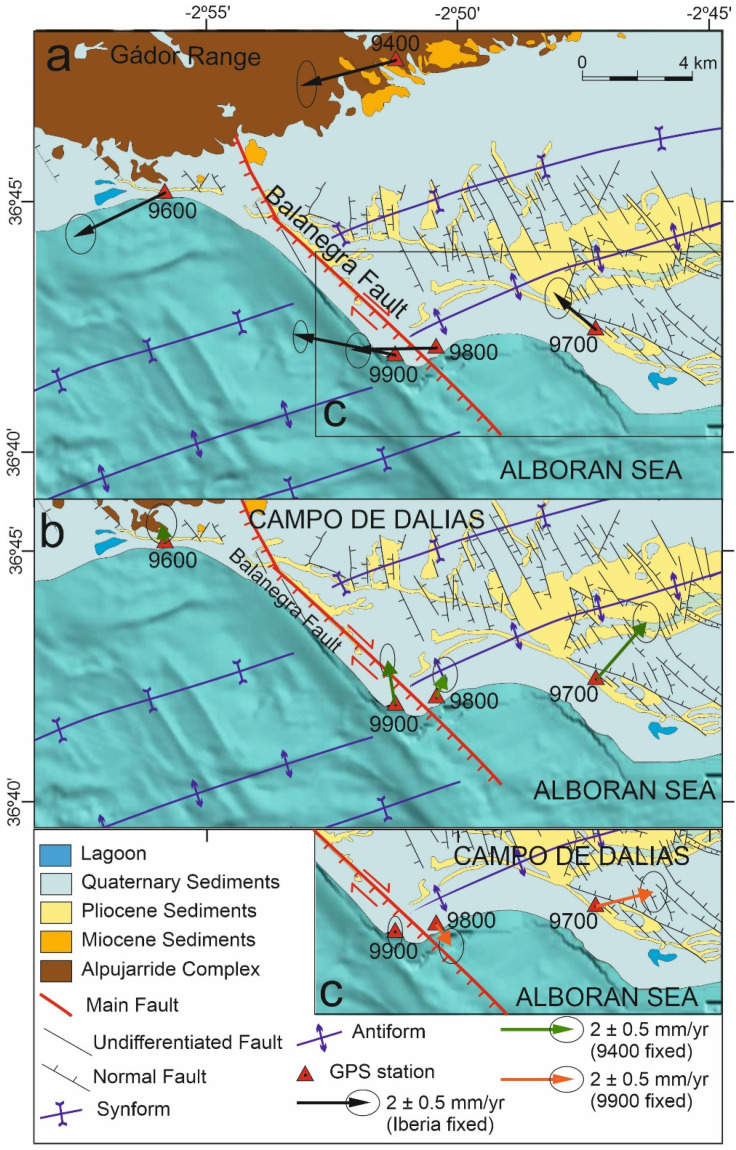
Geological sketch of the western Campo de Dalias, including residual GNSS velocities; error ellipses of 95% confidence: (**a**) Velocities with respect to stable Eurasia. (**b**) Velocities with respect to the relatively stable Site 9400 (Gador Range). (**c**) Velocities with respect to the relatively stable Site 9900 (southwest block of the Balanegra Fault).

The aim of this paper is to present the quantitative results of the western Campo de Dalias GNSS network (Figure 1 and Figure 2) in order to discuss the local interaction of folds and faults. This key area is located at the northern tip of the Betic–Alboran shear zone [18,19], where the wide fault zone affecting the Alboran Sea reaches the southern boundary of the Betic Cordillera (Figure 1). New GNSS data provide relevant findings about the present-day activity of the roll-back and indentation mechanisms proposed for the Gibraltar Arc’s development.

## 2. Geological Setting

The internal zones of the Betic and Rif Cordilleras comprise overprinted Alpine metamorphic complexes in the central part of the tectonic arc, including the Alboran Sea; they are separated by a flysch belt from the external zones that would correspond to the former Mesozoic South Iberian and African paleomargins. The Neogene–Quaternary sedimentary basins—including the Guadalquivir foreland basin, the intramontane basins, and the Alboran Sea—are filled by sediments and, locally, volcanic rocks. The main present-day reliefs, formed since the Tortonian [20,21], are a consequence of the interaction of folds and faults [22] whose activity continues and is related to present-day seismicity.

Seismic activity in the Eurasia–Africa plate boundary setting occurs along a section over 300 km wide, affecting the Betic and Rif Cordilleras, although the most intense activity can be linked to some specific sectors (Figure 1). One relevant seismic sector is that of a strip from the Al Hoceima area, crossing the Alboran Sea up to the Campo de Dalias, and formerly designated as the Trans-Alboran shear zone [23,24,25,26]. More precisely, according to the structures that remained active, the Betic–Alboran shear zone should be underlined (Figure 1) [18,19]. This fault zone was responsible for the seismicity in 1992 to 1994 that affected the Al Hoceima area and the Campo de Dalias, the catastrophic earthquake of Al Hoceima in 2004, and the seismic series of the southern Alboran Sea in 2016 [27]. The zone, including Al Idrissi and new faults, constitutes the western boundary of the crustal indenter that is recognized in the Alboran Sea [28], and continues eastward to join the Eastern Betic shear zone [29].

The controversial geological and geophysical data for this complex region have led to different tectonic concepts of the development of the Gibraltar Arc since Cenozoic times. Discussion remains alive, offering models mainly based on delamination [30,31] or subduction [32,33]; some include roll-back [13,14,34,35,36], or can be understood as hybrid models [37]. Moreover, the central Alboran Sea [28] and the eastern Betics [38,39] are affected by indentation tectonics that might contribute to the escape tectonics affecting the westernmost Gibraltar Arc [40]. Recent GNSS regional data (Figure 1) [13,14,15] provide a new framework for interpreting present-day tectonic movements and discussing their possible underlying processes. GNSS data on the central and western Betic Cordillera, for example, demonstrate that subduction with roll-back may be active at present in the western Gibraltar Arc [14,35,36]. In contrast, the kinematics of recent deformations along the eastern Betic Cordillera—i.e., affected by the Eastern Betic shear zone [41]—could be attributed to indenter tectonics. To date, however, we lack evidence of the interaction of these two active tectonic mechanisms proposed in the boundary regions.

The Campo de Dalias is a coastal area marking the transition from the southern Betic Cordillera, with a thickened crust, to the Alboran Sea, floored by a thinned continental crust [42]. This region of the internal zones of the Betic Cordillera has an Alpujarride metamorphic basement (marbles, phyllites, and schists) covered by late-Tortonian to Quaternary shallow marine and continental sediments [43]. The area is deformed by a fracture system comprising north–south- to northwest–southeast-faulted extensional and reactivated hybrid joints that accommodate the east-northeast–west-southwest extension [43]. Moreover, east-northeast–west-southwest folds developed since the Tortonian [44,45] accommodate the north-northwest–south-southeast shortening related to Eurasia–Nubia convergence.

## 3. GNSS Equipment and Data Processing

The western Campo de Dalias GNSS local network includes five sites (9400, 9600, 9700, 9800, and 9900; Figure 2, Table 1), and belongs to a wide regional network [13] built in 2006 and adequately conserved up to now. The network features self-centering mounting devices anchored to rocks; up to 2010 the measurement equipment comprised LEIAX1202 antennas and Leica Geosystem GX1230 receivers, yet since then LEIAR10 antennas and LEICA Geosystem AR10 receivers have been used. Records reflect at least 96 h for each observation, divided into 24 h and 30 s RINEX file sampling intervals.

**Figure 3:**
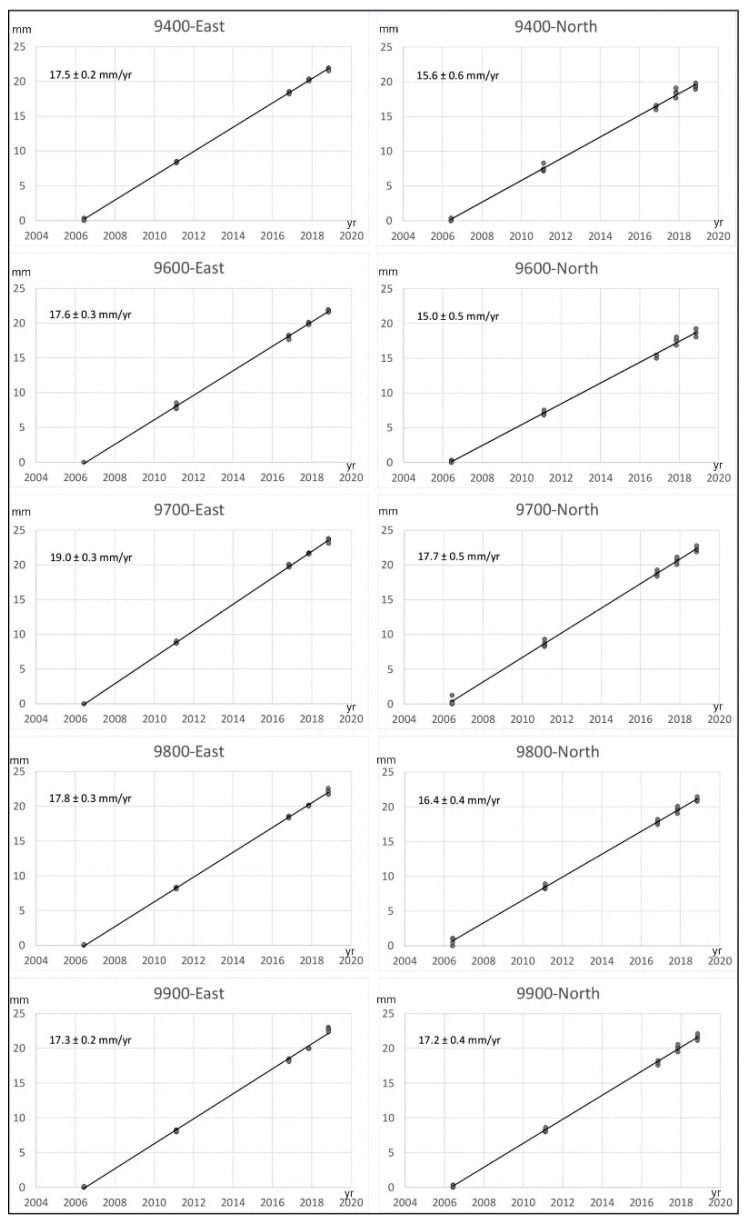
Position time series of the Campo de Dalias network stations (north and east components in millimeters). Absolute velocities are included in Table 1.

Data processing was done with GIPSY-OASIS software using JPL’s orbit and clock products for the GPS constellation [46]. Antenna calibration file, GMF troposphere mapping function, ionosphere TEC values, FES2004 ocean tide loading model [47], and WahrK1 terrestrial tide model were taken into account. Positions were computed according to 300 s sampling of the RINEX observations for the coordinate estimation in the IGS14 reference frame. The linear trend on the position time series (Figure 3) was computed using CATS time series analysis software [48]. The model applied to the original time series consists of an intercept, a site rate, and an offset to account for the change of antenna and receiver between the 2011 and 2016 campaigns. Finally, the residual velocity field was calculated with respect to stable Eurasia by subtracting the rigid motion of Eurasia from the ITRF2014 plate motion model [49] (Figure 2a). The plate velocity v_i_ of a site at position R_i_ on a plate with rotation described by the angular velocity vector of the plate Ω is given by the vector cross-product v_i_ = Ω × R_i_. To highlight the activity of local structures, we also determined relative velocities with respect to Sites 9400 (Figure 2b) and 9900 (Figure 2c).

## 4. GNSS Network Results

The GNSS network and position time series (Figure 2 and Figure 3) reveal a displacement pattern evidencing present-day progressive deformation of the region. All of the sites have a westward component of displacement with respect to stable Eurasia, in agreement with the regional GPS results (Figure 1) [15]. At a local scale, the approximately similar behavior of the two northern sites (9400 and 9600; Figure 2a) would suggest that they should be considered as being located roughly in the same tectonic block, practically unaffected by the present-day activity of the Balanegra Fault, and determinant of its northern tip.

When Site 9400 is taken as a stable reference for the northern region (Figure 2b), Site 9600 shows a short displacement towards the north-northwest, while Site 9900 shows marked displacements towards the north-northwest and Site 9800 is displaced towards the north-northeast. This displacement pattern evidences shortening related to east-northeast–west-southwest fold activity. In turn, Site 9700 displays a relative northeastward displacement, whose northward component could also be compatible with the shortening related to folds.

Finally, in order to analyze the horizontal deformation related to the Balanegra Fault near the coastline, the relative displacement of the network fixing Site 9900 may be considered (Figure 2c). In this framework, Site 9800 has a relative displacement to the southeast, evidencing the dextral activity of the Balanegra Fault close to the coastline. Moreover, the east-northeastward relative displacement of Site 9700 is consistent with east-northeast–west-southwest active extension related to the north-northwest–south-southeast normal fault set and the northwest–southeast Balanegra Fault, over an area probably affected by active fractures not yet identified at the surface.

## 5. Recent Offshore Active Tectonic Deformations West of the Campo de Dalias

**Figure 4:**
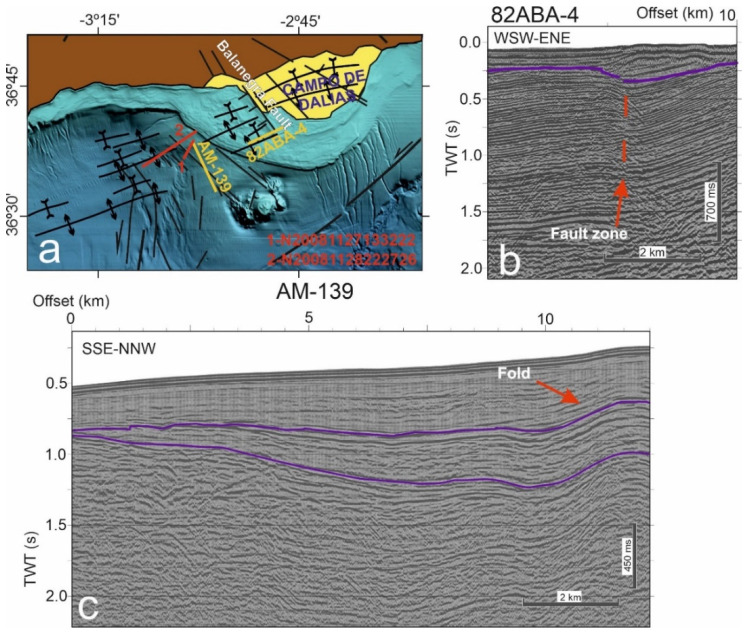
Seismic reflection profiles offshore of the Campo de Dalias: (**a**) Location of the profiles on the regional sketch of the main tectonic structures. (**b**) Profile 82 ABA-4, at the southern tip of the Balanegra Fault. (**c**) Profile AM-139, orthogonal to the east-northeast–west-southwest folds.

**Figure 5:**
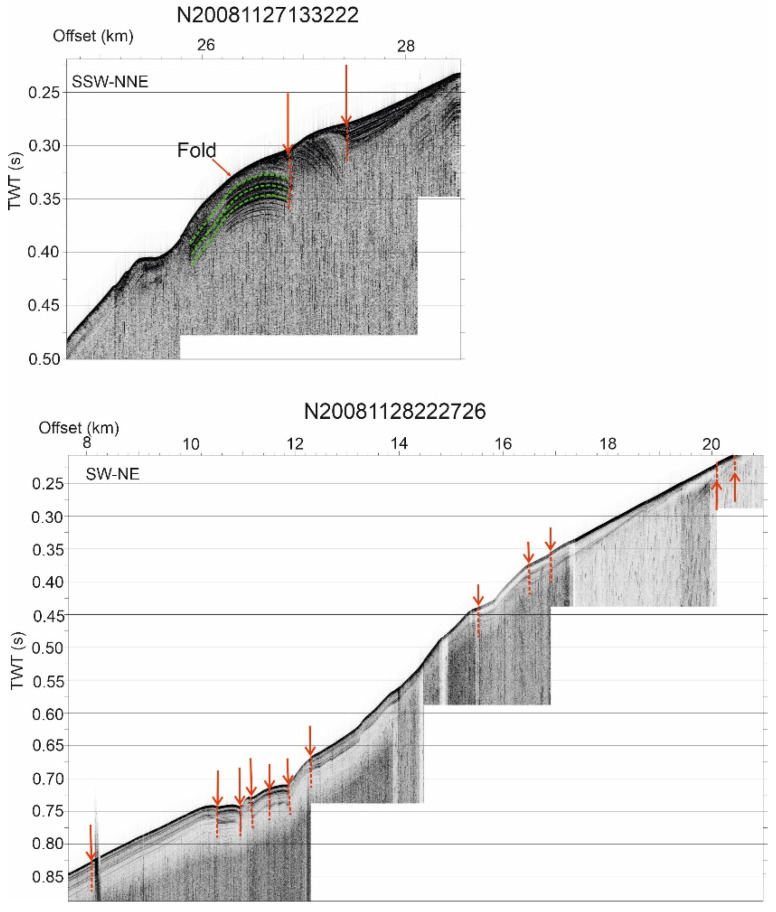
High-resolution reflection profiles west of the Campo de Dalias showing the very recent folds and faults (red arrows). Location in Figure 4.

Seismic reflection data offshore of the western Campo de Dalias (Figure 4 and Figure 5) were analyzed to determine the main features of the active faults and folds. Multichannel and high-resolution seismic reflection profiles from the database of the Instituto de Ciencias del Mar (ICM-CSIC, http://gma.icm.csic.es/sites/default/files/geowebs/OLsurveys/index.htm, accessed on 1 February 2022) were obtained following standard procedures of acquisition and processing. The southeastward offshore continuity of the Balanegra Fault zone (Figure 4a) is intersected orthogonally by profile 82 ABA-4 (Figure 4b), imaging a roughly vertical deformation zone, with a downthrown eastern block covered by unfaulted sediments with an eastward upraised bathymetric elevation. This geometry may be compatible with an early strike-slip motion that became inactive in this section. Profile AM-139 (Figure 4c), orthogonal to the east-northeast–west-southwest fold system, shows that folds are open and irregular, reaching kilometric sizes, yet deformation increases in depth, supporting the progressive activity. High-resolution profiles to the west of the Balanegra Fault (Figure 5) show that both folds and faults also affect the most recent sediments, even displacing the topography, and should therefore be considered to be active structures.

## 6. Discussion

**Figure 6:**
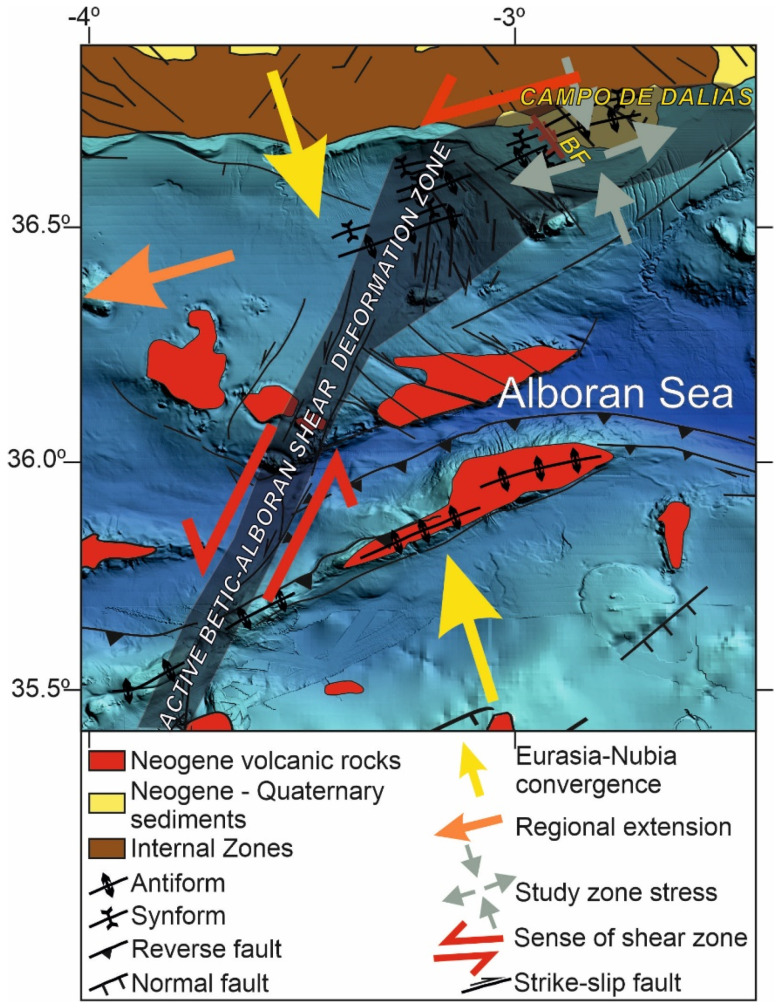
Tectonic sketch of the central Alboran Sea, relating deformation of the Campo de Dalias to the northeast tip of the active Betic-Alboran shear zone. BF: Balanegra Fault.

GNSS observations shed new light on active tectonics at a local scale in the western Campo de Dalias, and at a regional scale in the westernmost Mediterranean (Figure 1, Figure 2 and Figure 6). Detailed analysis of the Balanegra Fault, considered to be a simple normal fault, proves its complexity; it is a 10 km long fault, whose northwest tip lies near the Gádor Range boundary, while the southeast tip lies offshore (Figure 2). Seismic data on the activity of the Balanegra Fault are apparently controversial, pointing to dextral strike-slip earthquake focal mechanisms for northwest–southeast faults in the region [50], but normal slip during the 1993–94 seismic crisis [51]. In addition, previous high-precision leveling data and field geological research support the hypothesis that this fault has a vertical component with a westward downthrown block [17]. The present-day displacement, based on GNSS data, links these results, indicating that the Balanegra Fault is active, with a dextral creep displacement of close to 1 mm/year at the surface. This dextral displacement for the northwest–southeast fault is consistent with a deformation field determined by the north-northwest–south-southeast shortening simultaneous with east-northeast–west-southwest extension. Such kinematics can be found in other northwest–southeast faults outcropping in the Campo de Dalias [43], interpreted as shear or hybrid joints reactivated as faults under east-northeast–west-southwest extension. These results suggest that the fault has a behavior including creep at shallow levels, decreasing the seismic hazard, while its activity at depth represents moderate seismicity.

Furthermore, the southwestward motion of the northern sites (9400 and 9600), in contrast to the remarkable northwestward displacements of the southern sites (9700 and 9900) (Figure 2a), indicates regional north-northwest–south-southeast shortening, in agreement with the development of east-northeast–west-southwest folds that are still active. This small region—located above a sharp boundary between the Betic Cordillera’s thick continental crust and the Alboran Sea’s thin continental crust [42]—accommodates approximately 1 mm/year of the total 4.5–5 mm/year shortening of the Eurasian and Nubian plates. These folds have progressively developed since the Tortonian [44,45], owing to the north-northwest–south-southeast plate convergence. In this context, the Campo de Dalias, undergoing simultaneous northwest–southeast compression and northeast–southwest extension, may constitute an accommodation zone of the main north-northeast–south-southwest sinistral active fault zone that crosses the Alboran Sea—the Betic–Alboran shear zone, in the frontal part of the Alboran Sea indenter (Figure 6).

**Figure 7:**
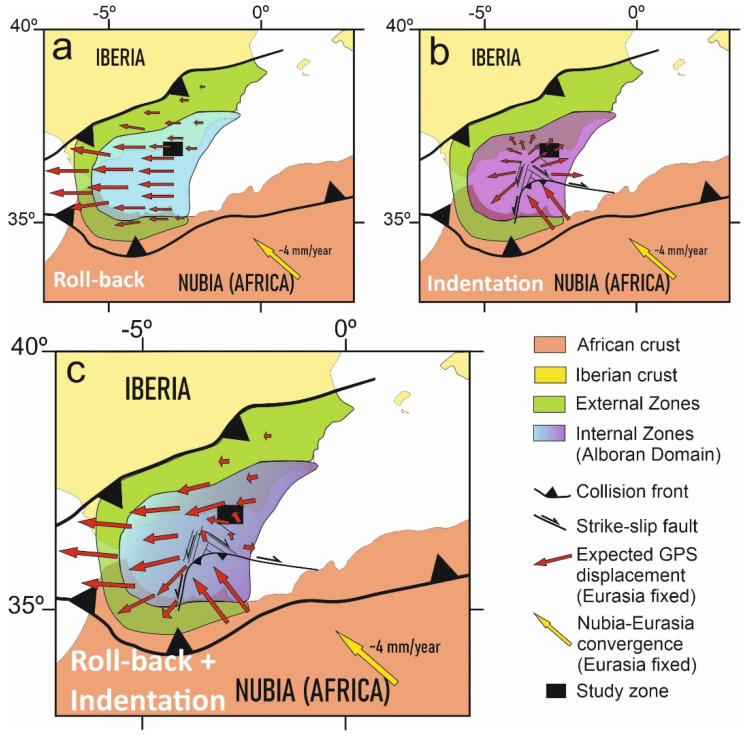
Expected displacement patterns during the development of the Gibraltar Arc with respect to stable Eurasia by (**a**) roll-back tectonics, with a regional westward displacement increasing toward the central and western parts of the tectonic arc; (**b**) indentation tectonics, with a radial pattern; and (**c**) the interaction of roll-back and indentation tectonics—in agreement with the Campo de Dalias GNSS network’s present results, the residual velocity field decreases and rotates clockwise from the north towards the south of the study area.

Although the entire region undergoes westward displacements (Figure 2a)—most likely a consequence of roll-back tectonics [35,36] (Figure 7)—the decreasing westward displacement towards the southern sites (Figure 2a) offers clues as to the tectonic mechanisms active in the westernmost Mediterranean (Figure 7). If a simple roll-back model is considered for the development of the Alboran Sea, increasing westward displacement towards the central Alboran Sea (Figure 7a) should be expected [35,36], but this is not observed. If simple indentation tectonics are envisaged for the central Alboran Sea, similar to the eastern Betic Cordillera indenters [41], a radial pattern of displacements should occur which, again, is not observed (Figure 7b). Thus, a model involving roll-back in the western Alboran Sea and indentation in the central and eastern areas would be needed to fit the observed displacement pattern, where the Campo de Dalias constitutes a boundary region (Figure 7c).

## 7. Conclusions

Despite the long periods necessary to yield suitable results in regions with slow deformation, GNSS network observations provide independent data that improve our geological and geophysical understanding of active tectonic structures. The GNSS Campo de Dalias network shows that the entire region is affected by continuous deformation, including north-northwest–south-southeast convergence accommodated by east-northeast–west-southwest folds, and orthogonal east-northeast–west-southwest extension accommodated by normal faults.

The GNSS results highlight the activity of the north-northwest–south-southeast normal Balanegra Fault, which constitutes a complex structure of 10 km in length, south of the Gádor Range, with a shallow dextral creep displacement of up to 1 mm/year, and a deep seismic character.

At a regional scale, the study area accommodated deformation of the north-northeast tip of the sinistral Betic–Alboran shear zone, crossing the Alboran Sea. The pattern of active deformation, with westward displacement decreasing toward the south, is consistent with the coexistence in this region of roll-back tectonics—a driving process well developed in the western Gibraltar Arc—and indentation tectonics, dominant farther east and south. These results point to the coexistence of different tectonic mechanisms during the development in the westernmost Mediterranean, leading to the development of the Gibraltar Arc.

## Figures and Tables

**Table 1:** Absolute velocity field in IGS14 reference frame and residual velocity field with respect to stable Eurasia based on the ITRF2014 PMM.

Site ID	Velocity (mm yr^−1^)		Uncertainty (mm yr^−1^)		Residual Velocity (mm yr^−1^)	
	East	North	East	North	East	North
940	17.5	15.6	±0.3	±0.7	−2.7	−0.9
960	17.6	15.0	±0.4	±0.6	−2.5	−1.6
970	19.0	17.7	±0.4	±0.6	−1.2	1.2
980	17.8	16.4	±0.4	±0.5	−2.4	−0.1
990	17.3	17.2	±0.3	±0.5	−2.9	0.7

## Data Availability

The data are included in Table 1 of this paper.
